# Type of Anemia, Chronic Non-cardiovascular Illnesses, and Outcomes of Patients with ST-segment Elevation Myocardial Infarction

**DOI:** 10.5041/RMMJ.10397

**Published:** 2020-04-29

**Authors:** Tomer Menzely, Robert Zukermann, Faheem Shehadeh, Rabia Shekh Muhammad, Doron Aronson, Michael Kapeliovich, Arthur Kerner, Sergey Yalonetsky, Lior Gepstein, Eugenia Nikolsky

**Affiliations:** 1 The Ruth and Bruce Rappaport Faculty of Medicine, Technion–Israel Institute of Technology, Haifa, Israel; 2 Department of Cardiology, Rambam Health Care Campus, Haifa, Israel

**Keywords:** Anemia, chronic illness, mean corpuscular volume, ST-segment elevation myocardial infarction

## Abstract

**Objectives:**

To assess the impact of different types of anemia and of concomitant non-cardiovascular chronic illnesses on outcomes of patients with ST-segment elevation myocardial infarction (STEMI) and baseline anemia admitted to the Intensive Cardiac Care Unit.

**Methods:**

Based on the mean corpuscular volume, anemia was stratified into: microcytic (<80 fL), normocytic (≥80, <96 fL), and macrocytic (≥96 fL). Data on concomitant chronic non-cardiovascular illnesses including malignancies were carefully collected. Endpoints included in-hospital bleeding as well as all-cause mortality at long-term follow-up.

**Results:**

Of 1,390 patients with STEMI, 294 patients had baseline anemia (21.2%), in whom normocytic, microcytic, and macrocytic anemia was present in 77.2%, 17.0%, and 5.8% patients, respectively. In-hospital bleeding occurred in 25 (8.5%) of the study population without significant differences between the three groups. At a mean follow-up of 5.5±3.5 years, 104 patients (35.4%) had died. Mortality was the highest in patients with macrocytic anemia, followed by patients with normocytic anemia and microcytic anemia (58.8%, 37.0%, and 20.0%, respectively; *P*=0.009). Chronic non-cardiovascular condition was identified as an independent predictor of both in-hospital bleeding (odds ratio=2.57, *P*=0.01) and long-term mortality (hazard ratio [HR] 1.54, *P*=0.019). Performance of coronary angiography within index hospitalization was associated with lower long-term mortality (HR 0.38, *P*=0.001). Mean corpuscular volume did not predict either in-hospital bleeding or mortality.

**Conclusions:**

Chronic non-cardiovascular illnesses are highly prevalent among patients with STEMI and baseline anemia, and are strongly associated with higher in-hospital bleeding and long-term mortality. Type of anemia is not related to prognosis post-STEMI.

## INTRODUCTION

Anemia is a frequent condition in patients hospitalized for acute coronary syndrome (ACS). In the contemporary trials and observational studies, anemia at baseline was present in up to one-quarter of the patients and consistently correlated with increased mortality.^1^–^4^ The reasons for negative impact of anemia on survival of patients with ACS are not known. Several mechanisms have been suggested, including excess of bleeding complications, overactivation of sympathetic nervous system, increased inflammatory response, negative impact of blood product transfusions, association with impaired renal function, and less frequent use of pharmacological agents recognized to improve survival post ACS.^2^,^5^–^8^

However, anemia is just a general laboratory abnormality with distinctive morphological characteristics of erythrocytes depending on the specific pathological conditions. Anemia develops by numerous pathophysiological pathways in patients with a wide range of chronic non-cardiovascular diseases, each of which may have a different impact on survival.^9^ Despite this, previous studies did not consider the possible impact of different types of anemia and/or of concomitant non-cardiovascular chronic illnesses on outcomes of patients with ACS.

Mean corpuscular volume (MCV), the average volume of red cells in a specimen, is a useful index to classify the type of anemia based on red cell morphology.^10^ The purpose of this study was to assess clinical outcomes and their relationship with MCV and chronic non-cardiovascular illnesses in consecutive patients with ST-segment elevation myocardial infarction (STEMI) and baseline anemia, included into the prospective registry.

## METHODS

### Study Population

All patients admitted to the Intensive Cardiac Care Unit at Rambam Health Care Campus in Haifa, Israel, with STEMI between 2006 and 2016 were included into the study. The analyzed data contained demographic, clinical, laboratory, and angiographic characteristics, as well as information on treatment strategies and medications at discharge. Data on concomitant chronic non-cardiovascular illnesses including malignancies were carefully collected for each patient.

Anemia was defined using World Health Organization criteria.^11^ Based on the MCV, anemia was stratified into microcytic (<80 fL), normocytic (≥80, <96 fL), and macrocytic (≥96 fL).^10^ Chronic non-cardiovascular illness was defined as a condition that lasts ≥1 year and requires ongoing medical attention and/or limits activities of daily living.^12^ Chronic kidney disease (CKD) was defined as having an estimated glomerular filtration rate (eGFR) of ≤60 mL/min/1.73 m^2^ as calculated at baseline by the Modification of Diet in Renal Disease (MDRD) equation.^13^ Peripheral arterial disease was defined as a history of intermittent claudication or lower-extremity vascular intervention (percutaneous or surgical), a history of stroke or transient ischemic attack, and/or prior carotid endarterectomy. Bleeding was defined as intracranial hemorrhage, gross hematuria, hematemesis, melena, bleeding requiring transfusion of red blood cells, retroperitoneal bleeding, and access site hematoma necessitating ultrasound evaluation and/or invasive management.

Analyzed clinical outcomes included rates of in-hospital bleeding as well as 1-year and long-term mortality. Data on survival were obtained from the Israeli National Population Register. Percutaneous coronary intervention (PCI) was performed using standard techniques. The study was conducted and approved by the Institutional Review Board of the hospital. Written informed consent was obtained from each patient.

### Statistical Analysis

Continuous variables are expressed as mean±SD, and categorical data are presented as frequencies. Differences among studied groups were compared using analysis of variance for continuous variables and chi-square statistics for categorical variables.

Multivariable analysis of predictors of in-hospital bleeding was performed using logistic regression with stepwise selection with entry and exit criteria of *P*<0.1. Two-sided 95% confidence interval (CI) was constructed around each point estimate of odds ratio (OR); *P*-value ≤0.05 was considered statistically significant.

Survival was estimated by the Kaplan–Meier method and compared by log-rank test. Independent predictors of mortality at 1 year and at long-term follow-up were performed using Cox proportional hazards regression with stepwise selection using entry and exit criteria of *P*<0.1 and adding clinically meaningful variables. The candidate variables introduced into the model included age, gender, diabetes mellitus, peripheral arterial disease, chronic non-cardiovascular illness, atrial fibrillation (paroxysmal, persistent, or permanent), Killip class on admission, performance of coronary angiography during index hospitalization, as well as baseline hemoglobin, MCV, and eGFR. All analyses were 2-sided, and significance was established at the 0.05 level.

## RESULTS

Of 1,390 patients with STEMI during the study period, 294 patients had baseline anemia (21.2%). Most patients with anemia had normocytic anemia (*n*=227; 77.2%), followed by microcytic anemia (*n*=50; 17.0%) and macrocytic anemia (*n*=17; 5.8%). Baseline clinical characteristics of the patients stratified by type of anemia are shown in Table 1. Patients with macrocytic anemia were older, had lower mean values of body mass index (BMI), baseline hemoglobin, and eGFR, and tended to have higher prevalence of atrial fibrillation. Other clinical characteristics were well matched between the groups, including prevalence of diabetes, peripheral arterial disease, chronic non-cardiovascular illnesses, prior myocardial infarction, PCI and/or coronary artery bypass grafting, as well as rates of anterior location of infarction and Killip class on admission. Chronic non-cardiovascular conditions were most frequently represented by gastrointestinal/hepatic (33.1%), respiratory (26.8%), connective tissue (10.2%), and active malignant (29.9%) illnesses. More than one chronic non-cardiovascular condition was present in 14.9% of the patients.

**Table 1:** Baseline Clinical Characteristics.

Characteristic	All Patients (*n*=294)	Microcytic Anemia (*n*=50)	Normocytic Anemia (*n*=227)	Macrocytic Anemia (*n*=17)	*P* Value
Age (mean±SD), years	67.0±12.7	61.5±13.7	68.0±12.4	69.4±9.8	0.032
Male, *n* (%)	206 (70.1%)	32 (64.0%)	160 (70.5%)	14 (82.4%)	0.346
Hypercholesterolemia, *n* (%)	176 (59.9%)	30 (60.0%)	135 (59.5%)	11 (64.7%)	0.914
Hypertension, *n* (%)	174 (59.2%)	31 (62.0%)	132 (58.2%)	11 (64.7%)	0.787
Diabetes mellitus, *n* (%)	115 (39.1%)	23 (46.0%)	84 (37.0%)	8 (47.1%)	0.393
Current smoking, *n* (%)	89 (30.3%)	18 (36.0%)	66 (29.1%)	5 (29.4%)	0.785
History of myocardial infarction, *n* (%)	62 (21.9%)	11 (22.0%)	46 (20.3%)	5 (29.4%)	0.876
History of percutaneous coronary intervention, *n* (%)	66 (22.4%)	13 (26.0%)	48 (21.1%)	5 (29.4%)	0.829
History of coronary artery bypass grafting, *n* (%)	13 (4.4%)	1 (2.0%)	12 (5.3%)	0 (0.0%)	0.390
Peripheral arterial disease, *n* (%)	45 (15.3%)	7 (14.0%)	34 (15.0%)	4 (23.5%)	0.563
Chronic kidney disease, *n* (%)	45 (15.3%)	6 (12.0%)	34 (15.0%)	5 (29.4%)	0.218
Congestive heart failure, *n* (%)	30 (10.2%)	4 (8.0%)	23 (10.1%)	3 (17.7%)	0.524
Chronic non-cardiac illness, *n* (%)	127 (43.4%)	18 (36.0%)	100 (44.7%)	9 (52.9%)	0.446
Malignancy, *n* (%)	38 (13.0%)	5 (10.0%)	28 (12.4%)	5 (29.4%)	0.104
Atrial fibrillation, *n* (%)	17 (5.8%)	2 (4.0%)	12 (5.3%)	3 (17.7%)	0.092
Anterior location of infarction, *n* (%)	132 (44.9%)	18 (36.0%)	107 (47.1%)	7 (41.2%)	0.340
Killip class at admission, *n* (%)					0.583
I	236 (80.3%)	44 (88.0%)	178 (78.4%)	14 (82.3%)	
II	32 (10.9%)	2 (4.0%)	28 (12.3%)	2 (11.8%)	
III/IV	26 (8.8%)	4 (8.0%)	21 (9.2%)	1 (5.9%)	
Body mass index (mean±SD), kg/m^2^	27.1±4.3	27.5±4.8	27.3±4.2	24.6±3.4	0.016
Baseline serum creatinine (mean±SD), g/dL	1.26±1.02	1.00 ±0.35	1.28±1.03	1.80±1.77	0.017
Estimated GFR (mean±SD), mL/min/1.73 m^2^	76.5±36.5	90.1±49.9	74.6±32.5	61.6±30.1	0.005
Estimated GFR ≤60 mL/min/1.73 m^2^, *n* (%)	99 (33.7%)	11 (22.0%)	81 (35.7%)	7 (41.2%)	0.14
Baseline hemoglobin (mean±SD), g/dL	11.6±1.1	11.1±1.4	11.8±8.8	10.6±1.7	<0.001
Mean corpuscular value (mean±SD), fL	86.2±7.0	75.6±4.6	87.5±4.0	100.6±4.2	<0.0001

GFR, glomerular filtration rate.

**Figure 1:**
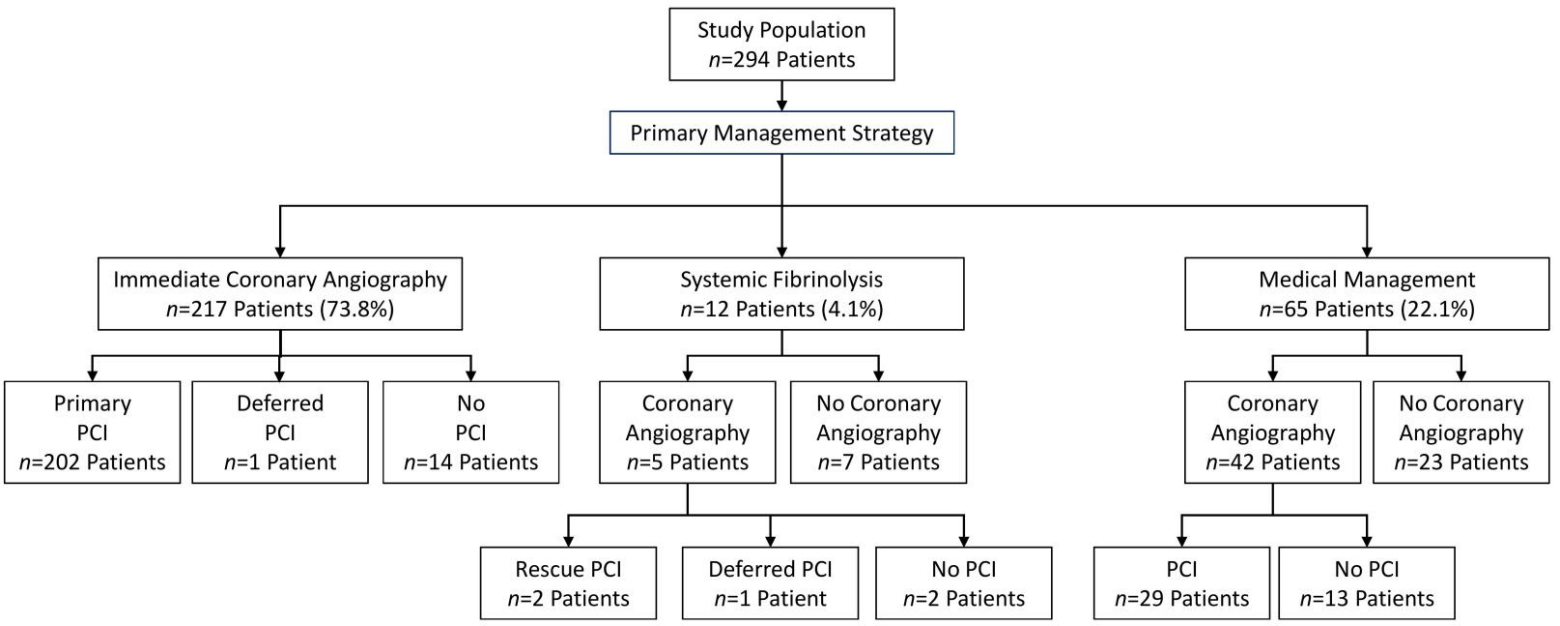
Initial Treatment Strategy of STEMI Patients with Anemia Flow chart of the initial treatment strategy of patients with STEMI and baseline anemia. PCI, percutaneous coronary intervention.

On admission, the initial strategy in most patients was reperfusion therapy (229 of 294; 77.9%) including 217 patients (73.8%) triaged to urgent coronary angiography and primary PCI if feasible, and 12 patients (4.1%) assigned to systemic fibrinolytic therapy (Figure 1 and Table 2). A total of 65 patients (22.1%) were triaged to non-reperfusion medical therapy. There were no significant differences in the treatment strategies between the groups. Among 217 patients who underwent urgent coronary angiography, primary PCI was successfully performed in 202 patients (93.1%). Among 12 patients who received fibrinolytic therapy, 5 underwent coronary angiography within index hospitalization, of whom 2 patients had rescue PCI for failed pharmacological reperfusion, 1 patient had deferred PCI, and 2 patients had no PCI. Among 65 patients who were initially triaged to medical therapy, deferred coronary angiography was performed in 42 patients (64.6%). Among a total of 264 patients who underwent coronary angiography (either as an initial strategy, or as a rescue, or deferred) PCI was not performed in 29 patients (11.0%) (14, 2, and 13 patients, respectively). Reasons for not performing PCI in these patients included the inability to ascertain the culprit lesion and/or lack of significant stenosis in 12, anatomy considered not suitable for revascularization or culprit vessel supplying a small amount of myocardium in 12, and unspecified reasons in 5 patients.

**Table 2:** Procedures and Treatment of Patients.

Characteristics	All Patients (*n*=294)	Microcytic Anemia (*n*=50)	Normocytic Anemia (*n*=227)	Macrocytic Anemia (*n*=17)	*P* Value
Urgent coronary angiography, *n* (%)	217/294 (73.8%)	40/50 (80.0%)	164/227 (72.3%)	13/17 (76.5%)	0.788
Primary PCI, *n* (%)	202/217 (93.1%)	38/40 (95.0%)	152/164 (92.7%)	12/13 (92.3%)	0.512
Door-to-balloon time (mean±SD), min	85.6±79.6	66.5±40.3	88.2±79.0	112.1±79.6	0.16
Intravenous fibrinolysis, *n* (%)	12/294 (4.1%)	2/50 (4.0%)	9/227 (4.0%)	1/17 (5.9%)	0.928
Rescue PCI, *n* (%)	2/12 (16.7%)	0/2 (0.0%)	1/9 (11.1%)	1/1 (100.0%)	
No reperfusion, *n* (%)	65/294 (22.1%)	8/50 (16.0%)	54/227 (23.8%)	3/17 (17.7%)	0.776
Late coronary angiography, *n* (%)	42/65 (64.6%)	7/8 (87.5%)	34/54 (63.0%)	1/3 (33.3%)	
Angiographic features, *n* (%)					0.660
Single-vessel disease	109/264 (41.3%)	22/47 (46.8%)	80/202 (39.6%)	7/15 (46.7%)	
Double-vessel disease	76/264 (28.8%)	12/47 (25.5%)	62/202 (30.7%)	2/15 (13.3%)	
Triple-vessel disease	71/264 (26.9%)	11/47 (23.4%)	54/202 (26.7%)	6/15 (40.0%)	
Non-significant (<50%) stenosis or no stenosis	8/264 (3.0%)	2/47 (4.3%)	6/202 (3.0%)	0/15 (0.0%)	
Left main disease	8/264 (3.0%)	1/47 (2.1%)	7/202 (3.5%)	0/15 (0.0%)	0.694
Left ventricular ejection fraction (mean±SD), %	44.4±12.9	47.2±12.8	43.8±12.8	43.8±13.7	0.26

PCI, percutaneous coronary intervention.

**Figure 2:**
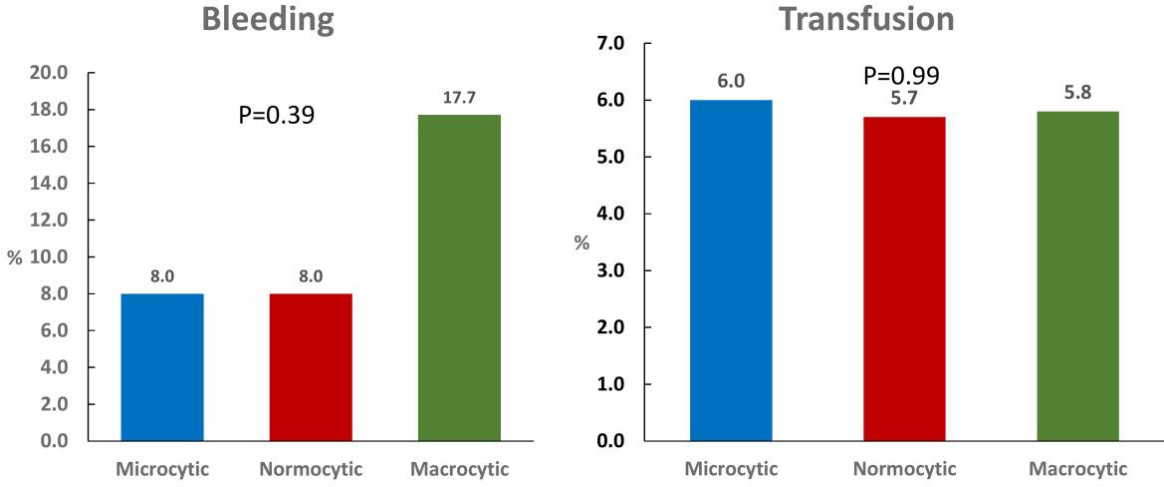
Bleeding and Transfusion in STEMI Patients with Anemia In-hospital bleeding and transfusion in patients with STEMI and baseline anemia in relation to mean corpuscular volume.

In-hospital bleeding events occurred in 25 (8.5%) of the study population, and 17 patients (5.8%) received red blood cell transfusion. Although patients with macrocytic anemia had significantly lower mean values of nadir hemoglobin compared to patients with microcytic and normocytic anemia (9.9± 1.8 g/dL versus 10.1±1.5 g/dL versus 10.8±1.3 g/dL, respectively; *P*=0.0008), there were no significant differences in incidence of bleeding and transfusion between the three groups (Figure 2). Rate of bleeding or transfusion did not differ significantly among patients who had, versus patients who did not have, coronary angiography during index hospitalization (12.6% versus 10.0%; *P*=0.701). Only 1 patient died within index hospitalization.

**Figure 3:**
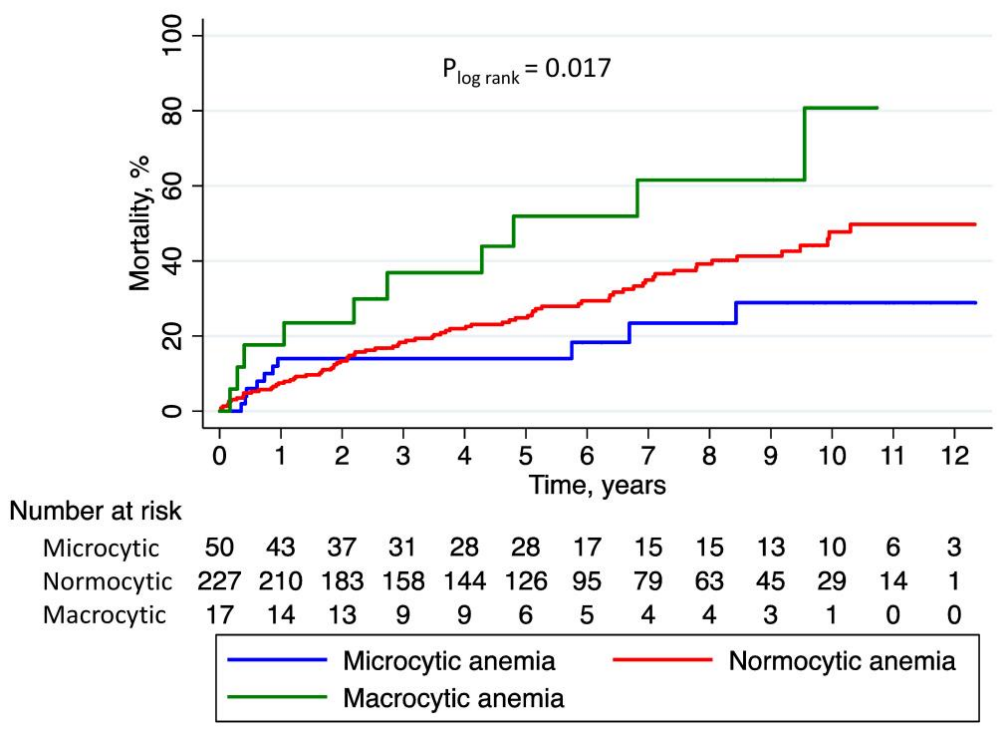
Long-term Mortality in STEMI Patients with Anemia Kaplan-Meier estimates of long-term mortality in patients with STEMI and baseline anemia stratified by mean corpuscular volume. PCI, percutaneous coronary intervention; STEMI, ST-elevation myocardial infarction.

At 1 year, 27 (9.2%) patients had died, including 7 (14.0%) patients with microcytic anemia, 17 (7.5%) patients with normocytic anemia, and 3 (17.7%) patients with macrocytic anemia (*P*=0.163). At a mean follow-up of 5.5±3.5 years, 104 patients (35.4%) had died. As shown in Figure 3, mortality at follow-up was the highest in patients with macrocytic anemia, followed by patients with normocytic anemia and microcytic anemia (58.8%, 37.0%, and 20.0%, respectively; *P*=0.009).

All but 3 patients (99.0%) were recommended aspirin at discharge (Table 3). Treatment with P2Y_12_ receptor inhibitor was not prescribed in 10.3% of the patients. Oral anticoagulation (mostly with vitamin K antagonist) was recommended to 6.5% of the patients, with the highest rates among patients with macrocytic anemia. Triple therapy (vitamin K antagonist plus dual antiplatelet therapy) was prescribed to 3.8% of the patients. One patient was discharged on warfarin only, and one patient was discharged neither on antiplatelet nor on antithrombotic medication (both in the group with normocytic anemia). Two patients with macrocytic anemia were discharged on clopidogrel plus warfarin.

**Table 3:** Medications at Discharge.

Medication	All patients (*n*=293*)	Microcytic Anemia (*n*=50)	Normocytic Anemia (*n*=226*)	Macrocytic Anemia (*n*=17)	*P* Value
Aspirin, *n* (%)	290 (99.0%)	50 (100.0%)	224 (99.1%)	16 (94.1%)	0.104
Aspirin monotherapy, *n* (%)	28 (9.6%)	5 (10.0%)	21 (9.3%)	2 (11.8%)	0.981
P2Y_12_ receptor inhibitor, *n* (%)	263 (89.7%)	45 (90.0%)	203 (89.8%)	15 (88.2%)	0.977
Oral anticoagulation, *n* (%)	19 (6.5%)	1 (2.0%)	15 (6.6%)	3 (17.7%)	0.076
Warfarin, *n* (%)	16 (5.5%)	1 (2.0%)	12 (5.3%)	3 (17.7%)	0.048
Novel oral anticoagulants, *n* (%)	3 (1.0%)	0 (0.0%)	3 (1.3%)	0 (0.0%)	0.550
Triple therapy, *n* (%)	11 (3.8%)	1 (2.0%)	9 (4.0%)	1 (5.9%)	0.715

* One patient died during index hospitalization.

Independent predictors of in-hospital bleeding included female gender (OR 4.34; 95% CI 1.49–12.65; *P*=0.007), presence of chronic non-cardiovascular illness (OR 2.57; 95% CI 1.25–5.26; *P*=0.01), lower eGFR (OR [per 10 mL/min/1.73 m^2^ decrease] 1.30; 95% CI 1.08–1.54; *P*=0.005), and lower BMI (OR 1.14; 95% CI 1.01–1.43; *P*=0.032). Independent predictors of mortality are presented in Table 4. One-year mortality was independently associated with diabetes mellitus, while long-term mortality was predicted by atrial fibrillation, older age, lower baseline hemoglobin, lower eGFR, and peripheral arterial disease. Chronic non-cardiovascular illness independently predicted both 1-year and long-term mortality, while performance of coronary angiography within index hospitalization was associated with lower mortality. The MCV predicted neither in-hospital bleeding nor mortality.

**Table 4:** Multivariable Predictors of 1-Year and Long-term Mortality.

Variable	Hazard Ratio	95% Confidence Interval	*Z* Coefficient	*P* Value
At 1 year:				
Diabetes mellitus	3.60	1.29–10.1	2.45	0.014
Coronary angiography	0.16	0.049–0.53	−3.01	0.003
Chronic non-cardiovascular illness	3.17	1.50–6.69	3.03	0.002
MCV	1.00	0.97–1.03	0.12	0.900
At long-term follow-up:				
Atrial fibrillation	3.50	1.75–6.70	3.55	<0.0001
Age (per 10 years increase)	1.44	1.15–1.82	3.18	0.001
Coronary angiography	0.38	0.22–0.68	−3.27	0.001
Hemoglobin at baseline (per 1 g/dL decrease)	1.33	1.06–1.69	−2.44	0.015
eGFR (per 10 mL/min/1.73 m^2^ decrease)	1.12	1.02–1.23	−2.20	0.015
Chronic non-cardiovascular illness	1.54	1.07–2.21	2.35	0.019
Peripheral arterial disease	1.72	1.07–2.77	2.22	0.026
MCV	0.96	0.90–1.03	−1.09	0.280

eGFR, estimated glomerular filtration rate; MCV, mean corpuscular volume

## DISCUSSION

The main results of the present analysis of consecutive patients with STEMI and baseline anemia are as follows: (1) The majority of patients (three-quarters) had normocytic anemia; (2) More than one-third of the patients with anemia had chronic non-cardiovascular illness or malignancy; (3) One-fifth of patients with anemia were declined reperfusion therapy on admission, and one-tenth of patients did not receive dual antiplatelet therapy at discharge; (4) Chronic non-cardiovascular conditions, and not the type of anemia, predicted occurrence of in-hospital bleeding and mortality both at 1-year and at long-term follow-up; and (5) Performance of coronary angiography within index hospitalization was independently associated with improved 1-year and long-term survival.

Our study confirms the high prevalence of baseline anemia among STEMI population (one-fifth of patients in this analysis), and suggests an explanation for the negative relationship between baseline anemia and mortality. Long-term survival in this high-risk population is driven not by the type of anemia but rather by a combination of older age, diabetes mellitus, peripheral arterial disease, impaired renal function, atrial fibrillation, and serious non-cardiovascular co-morbidities. While mortality was significantly associated with macrocytic anemia by univariate analysis, this relationship disappeared after multivariable adjustment including presence of non-cardiovascular illness(es). We analyzed aggregate non-cardiovascular illnesses separately from traditional cardiovascular conditions and CKD, which are typically considered in randomized trials and observational series. While diabetes, peripheral arterial disease, and CKD have been repeatedly demonstrated as factors worsening prognosis post-STEMI,^14^–^16^ the role of chronic non-cardiovascular illnesses has not been assessed in this scenario. Our analysis emphasizes the importance to include a broader spectrum of clinical information, which is clearly relevant to prognosis. Although it is not practical to adjust for all comorbid conditions, the use of quantitative indices as an aggregate comorbidity measure should be considered.^17^

Complex clinical scenarios coupled with unfavorable angiographic features in patients with baseline anemia pose therapeutic dilemmas for physicians and often result in low rates of contemporary therapies known to improve prognosis in the setting of STEMI, mainly the timely performance of coronary angiography followed by mechanical reperfusion. Actual occurrence of or concern on bleeding complications likely causes less frequent usage of dual antiplatelet therapy.

A remarkable finding of this analysis is that among patients with STEMI and baseline anemia there was no excess of bleeding events or transfusions in relation to performance of coronary angiography within index hospitalization. However, long-term mortality among patients who had coronary angiography was half that of patients who did not have coronary angiography (33.3% versus 68.2%, *P*<0.0001). Moreover, by multivariable analysis non-performance of coronary angiography predicted mortality both at 1 year and at long-term. This strongly indicates a benefit of timely myocardial reperfusion even in the complex scenario of STEMI and anemia.

A key limitation of this study is that the precise ways chronic illnesses impacted mortality cannot be elucidated, given a substantial heterogeneity of chronic conditions and their severity. Plausible reasons include progression of chronic non-cardiovascular illnesses, performance of invasive procedures necessitating discontinuation of antiplatelet medications, and possible side effects of medications to treat chronic conditions.

In conclusion, chronic non-cardiovascular illnesses are highly prevalent in populations with STEMI and baseline anemia. Regardless of the type of anemia, chronic non-cardiovascular conditions have a strong negative impact on long-term mortality post-STEMI. Performance of coronary angiography followed by revascularization when possible is associated with improved survival in patients with STEMI and anemia. Future clinical trials should collect detailed information on chronic non-cardiovascular illnesses to allow more accurate interpretation of outcomes and optimization of treatment strategies in these high-risk patients.

## Abbreviations

ACS: acute coronary syndrome
BMI: body mass index
CKD: chronic kidney disease
CI: confidence interval
eGFR: estimated glomerular filtration rate
HR: hazard ratio
MCV: mean corpuscular volume
OR: odds ratio
PCI: percutaneous coronary intervention
STEMI: ST-segment elevation myocardial infarction
